# Genome analysis of methicillin resistance in *Macrococcus caseolyticus* from dairy cattle in England and Wales

**DOI:** 10.1099/mgen.0.000191

**Published:** 2018-06-19

**Authors:** Alison C. MacFadyen, Elizabeth A. Fisher, Ben Costa, Cassie Cullen, Gavin K. Paterson

**Affiliations:** ^1^​ Royal (Dick) School of Veterinary Studies and The Roslin Institute, University of Edinburgh, Edinburgh, UK; ^2^​ School of Life Sciences, University of Hull, Kingston upon Hull, UK; ^3^​ School of Biology, University of St Andrews, St Andrews, UK

**Keywords:** macrococci, staphylococci, veterinary microbiology, methicillin resistance, mec genes

## Abstract

Species of the genus *Macrococcus* are widespread commensals of animals but are becoming increasingly recognised as veterinary pathogens. They can encode methicillin resistance and are implicated in its spread to the closely-related, but more pathogenic, staphylococci. In this study we have identified 33 isolates of methicillin-resistant *Macrococcus caseolyticus* from bovine bulk tank milk from England and Wales. These isolates were characterised to provide insight into the molecular epidemiology of *M. caseolyticus* and to discern the genetic basis for their methicillin resistance. Antimicrobial susceptibility testing was performed by Vitek2 and disc diffusion. Isolates were whole-genome sequenced to evaluate phylogenetic relationships and the presence of methicillin resistance determinants, *mecA*–*D*. All 33 isolates were phenotypically methicillin-resistant according to cefoxitin disc diffusion, cefoxitin Etest and oxacillin resistance assessed by Vitek2. In contrast only a single isolate was resistant in the Vitek2 cefoxitin screen. Twenty-seven isolates were positive for *mecD* and six were positive for *mecB. mecA* and *mecC* were not detected. The results of phylogenetic analysis indicated that these methicillin-resistant isolates represented a heterogeneous population with both *mecB* and *mecD* found in diverse isolates. Isolates had a widespread distribution across the sampled region. Taken together with the role of *M. caseolyticus* in veterinary infections, including bovine mastitis, and in the potential spread of methicillin resistance to more pathogenic staphylococci, this work highlights the need to better understand their epidemiology and for increased awareness among veterinary microbiology laboratories.

## Data Summary

1. All genome sequences generated in this study have been deposited in Genbank under Bioproject PRJNA420921, study accession number SRP126085. Accession numbers for individual isolates (Biosample, SRA and Assembly) are provided in Table S1 (available in the online version of this article).

**Impact Statement**

Commensal bacteria of animals can be important reservoirs of resistance determinants for dissemination to more pathogenic organisms. Their study may reveal important insights into the evolution and epidemiology of antimicrobial resistance, however, they often receive little attention. Macrococci are one such example with relatively little known about their molecular epidemiology and with very few publicly available genomes. This is despite an increasing recognition of macrococci as veterinary pathogens themselves and their potential role as a reservoir for methicillin-resistance determinants. To address this we used whole-genome sequencing and antimicrobial susceptibility testing to characterise a collection of 33 bovine isolates of methicillin-resistant *Macrococcus caseolyticus*. Our data indicates that the methicillin-resistance determinants *mecB* and *mecD* are present in a diverse bacterial population, spread widely in the study area. We find evidence for both local and more distant spread of related isolates. Furthermore, we demonstrate that highly-related isolates may carry either *mecB* or *mecD*. This is, to our knowledge, the largest collection of sequenced macrococci to date and the first detection of *mecB* and *mecD* in England and Wales. The data are important in understanding the epidemiology of *M. caseolyticus* itself as well as the resistance determinants *mecB* and *mecD*.

## Introduction

The genus *Macrococcus* consists of eight species primarily isolated from animal skin and food products such as milk and meat. These Gram-positive, catalase-positive bacteria are closely related to staphylococci and historically were included in that genus until being assigned to their own genus, *Macrococcus*, in 1988 [1]. Unlike staphylococci, macrococci are not considered to be common as pathogens. However, a role for *Macrococcus*, in particular *Macrococcus caseolyticus* and *Macrococcus canis* in veterinary infections has become increasingly recognised. *M. caseolyticus* having been isolated from ovine abscesses [2], bovine mastitis [3], canine dermatitis [4], canine rhinitis [5] and canine otitis [3] with *M. canis* being linked to a range of canine infections [4]. Methicillin resistance has been described in both *M. caseolyticus* and *M. canis*. Similarly to methicillin resistance in staphylococci [6], the basis for this resistance is a non-native penicillin-binding protein, PBP2a, encoded by a *mec* gene [3, 4, 7]. Four *mec* gene types, *mecA–D*, have been described among staphylococci and macrococci, a type being defined as sharing <70 % nucleotide identity to the others [8]. In the case of methicillin-resistant staphylococci, including methicillin-resistant *Staphylococcus aureus* (MRSA), *mecA* predominates, with *mecC* found in smaller numbers [9, 10]. Neither *mecA* or *mecC* have been reported in macrococci, instead the two other *mec* types, *mecB* and *mecD*, have been found in *M. caseolyticus* [3–5, 11] with *mecB* also being described in *M. canis* [4]. The relatedness of macrococci and staphylococci raises the potential for horizontal gene transfer between the genera and the spread of *mecB-* and *mecD*-mediated resistant to the more pathogenic staphylococci. Indeed the *mecB* gene complex in *M. caseolyticus* has been proposed as a possible primordial form of the *mecA* gene complex found in methicillin-resistant staphylococci [12]. Strong evidence for the cross-genus transmission of *mec* genes and methicillin resistance between macrococci and staphylococci comes from the discovery in a MRSA carriage isolate from a hospital inpatient of *mecB*, which was encoded on a plasmid related to a *mecB*-carrying plasmid from *M. caseolyticus* [13]. This isolate was further noteworthy as being the first description of plasmid-encoded methicillin resistance in staphylococci, raising the potential worry of the rapid dissemination of resistance [13]. Such *mecB*-positive isolates also pose a potential diagnostic problem with discordance between the phenotypic detection of methicillin resistance and negative results from the molecular-based detection of PBP2a or *mecA* and *mecC*. A further concern regarding the transfer of *mec* genes from macrococci to staphylococci is the potential for *mecD* to mediate resistance to the anti-MRSA cephalosporins, ceftobiprole and ceftaroline, as indicated from work done in *M. caseolyticus* [3]. Relatively little is known about the molecular epidemiology of macrococci. This is despite their role as pathogens, their possession of methicillin-resistance and the risk and the consequences of that resistance spreading to more pathogenic staphylococci. Here we provide insight into this subject with the characterisation, including whole-genome sequencing, of a collection of 33 methicillin-resistant *M. caseolyticus* isolated from dairy cattle bulk tank milk in England and Wales.

## Methods

### Isolate collection

Bulk tank milk samples collected in September 2015 and February 2016 from English and Welsh dairy farms were supplied by National Milk Laboratories (Chippenham, UK). Milk samples were processed for the detection of MRSA as described previously [14] except for the omission in this study of Staph Brilliance 24 as a medium after initial isolation on MRSA Brilliance (Oxoid). In addition to the 33 methicillin-resistant *M. caseolyticus* isolated in this work, the genome analysis included the two publicly available assembled *M. caseolyticus* genomes; *M. caseolyticus* IMD0819 (NZ_CP021058.1) [3] and JCSC5402 (NC_011999) [12].

### Antimicrobial susceptibility testing

Antimicrobial susceptibility testing was performed with a Vitek2 (BioMérieux) using the AST-P634 card following the manufacturer’s instructions. Cefoxitin disc diffusion was performed according to The European Committee on Antimicrobial Susceptibility Testing (EUCAST) methodology (version 6.0) with the MIC of cefoxitin determined using the Etest (BioMérieux). All interpretation of *M. caseolyticus* was done according to The European Committee on Antimicrobial Susceptibility Testing criteria for coagulase-negative staphylococci using *Staphylococcus aureus* NCTC6571 and NCTC12493 as control strains.

### Whole-genome sequencing and analysis

Whole-genome sequencing (WGS), using Illumina HiSeq technology with 2×250 bp paired-end reads, read trimming and assembly was performed by Microbes NG (University of Birmingham, UK). Reads were trimmed using Trimmomatic version 0.30 [15], using a sliding window quality cut-off of 15. Genome assembly was done *de novo* using SPAdes, version 3.7 [16], with default parameters for 250 bp Illumina reads. Assemblies were annotated by the NCBI Prokaryotic Genome Annotation Pipeline. The presence of *mec* gene types *mecA*, *mecB*, *mecC* and *mecD* was detected by blast and the arrangement of the *mec* gene complex visualized using Artemis. Based on the work of Arredondo-Alonso *et al.* [17] on the detection of plasmids within WGS data, it was decided to combine the method employed by PlasmidFinder [18] and plasmidSPAdes [19]. Briefly, PlasmidFinder determines the presence of a plasmid by identifying plasmid-associated replicon sequences within the WGS data, whereas plasmidSPAdes utilises assembly graphs produced by SPAdes, estimates chromosome coverage and assigns contigs with read contig coverage differing from the chromosome coverage as plasmid. As PlasmidFinder is optimised for enterobacterial genomes, plasmid replicon sequences from *M. caseolyticus* plasmids, pMCCL1 and pMCCL2 (accession number AP009485.1 and AP009486.1, respectively), were used to search contigs produced by plasmidSPAdes. To assign the *mec* genes as being encoded on a plasmid, plasmidSPAdes had to identify putative plasmid-associated contigs and the pMCCL1/2 replicon sequences had to be present within those contigs, as well as the *mec* gene of interest. Phylogenetic relationships among the isolates were assessed with SeqSphere+ software version 4.1.9 (Ridom) [20] using a core genome multilocus sequence typing (cgMLST) and accessory genome scheme based on all 35 isolates in this study with *M. caseolyticus* IMD0819 (NZ_CP021058.1) as the reference genome. Reference genome filters were a minimum length >50 bases, a start and stop codon at the beginning and end of the gene, a homologous gene filter (requiring no multiple copies of a gene with blast overlap >100 bp, identity >90.0 %), a gene overlap filter (requiring no overlap with other genes of more than four bases) and an exclude sequences filter (requiring no blast hit with overlap >100 bp, identity >90.0 % in excluded sequences). The query genome blast search required a blast hit with overlap=100 %, identity >90.0 % in every query genome using blast options: word size=11, mismatch penalty=−1, match reward=1, gap open costs=5, gap extension costs=2. A query genome stop codon percentage filter was applied requiring a single stop codon at end of gene in >80 % query genomes. Omitting missing values from isolates this resulted in a scheme for this study of 1550 gene targets consisting of 1415 cgMLST and 135 accessory genome targets.

### Nucleotide accession numbers

The sequencing reads and annotated assemblies generated in this study have been deposited in the NCBI database under Bioproject PRJNA420921, study accession number SRP126085 with individual accession numbers provided in in Table S1, available online.

## Results

### Isolation and antimicrobial susceptibility testing

Convenience sampling of dairy cattle bulk tank milk for methicillin-resistant staphylococci identified 33 isolates which grew on MRSA Brilliance agar, producing smooth, regular-edged, convex colonies coloured white or cream. Identification of these Gram-positive cocci was initially performed using Vitek2 and the GP identification card. This returned a variety of identifications with the majority being unidentified (14 isolates), along with five different staphylococcal species (ten isolates) and *Kocuria kristinae* (nine isolates), Table 1. These inconsistent results raised interest in this collection of resistant isolates, which were subsequently genome sequenced, revealing all 33 as being *M. caseolyticus*. None of the milk samples containing methicillin-resistant *M. caseolyticus* were positive for MRSA.

**Table 1.** Summary details of the 33 methicillin-resistant isolates of *M. caseolyticus* examined in this study

No.*	**Isolate**	**Vitek2 identification**	**Region**	**Date of collection**	**Antibiogram**†	**Cefoxitin MIC**	***mec* type**
1	5781_EF64	Unidentifed	Wiltshire	Sep-15	pen ox ICR ery clin‡	12	*mecD*
2	5786_EF153	*Staphylococcus simulans*	Cheshire	Feb-16	pen ox ery clin tet (trim)	32	*mecD*
3	5457_3–80	*Kocuria kristinae*	Cheshire	Sep-15	pen ox ery clin tet (trim)	32	*mecD*
4	5804_BC29	*K. kristinae*	Cheshire	Feb-16	pen ox FA (clin)	32	*mecD*
5	5194_2_25	*K*. *kristinae*	Cheshire	Sep-15	pen ox FA (clin)	128	*mecB*§
6	5818_BC116	*Staphylococcus hominis*	Cheshire	Feb-16	pen ox (clin)	16	*mecD*
7	5193_2_23	Unidentified	North Yorkshire	Sep-15	ox (clin)	12	*mecD*
8	5196_2_38	Unidentified	North Yorkshire	Sep-15	pen ox (clin)	8	*mecD*
9	5782_EF_83	Unidentified	Dorset	Sep-15	pen ox fox ery clin tet	94	*mecD*
10	5197_42554	Unidentified	Devon	Sep-15	pen ox clin	96	*mecD*
11	5789_EF199	*Staphylococcus saphrophyticus*	Devon	Feb-16	pen ox clin tet	64	*mecD*
12	5784_EF114	Unidentified	Devon	Sep-15	pen ox ery clin tet	32	*mecD*
13	5783_EF107	Unidentified	Gloucestershire	Sep-15	pen ox FA (clin)	64	*mecB*§
n/a	5452_CC83	Unidentified	Not applicable (milk tanker)	Feb-16	pen ox clin tet (trim)	12	*mecD*
14	5812_BC73	*K. kristinae*	Gwent	Feb-16	pen ox tet (clin)	192	*mecB*§
15	5814_BC75	*S. hominis*	Gwent	Feb-16	pen ox tet (clin)	>256	*mecB*§
16	5815_BC85	*K. kristinae*	Monmouthshire	Feb-16	pen ox tet	12	*mecD*
17	5816_BC109	Unidentified	Gwent	Feb-16	pen ox tet FA (clin)	128	*mecB*§
18	5813_BC74	*K.kristinae*	Abergavenny	Feb-16	ox tet FA (clin)	24	*mecD*
19	5459_5–49	*K. kristinae*	Cornwall	Sep-15	pen ox ery clin tet	32	*mecD*
20	5458_5–53	Unidentified	Cornwall	Sep-15	pen ox (clin)	32	*mecD*
21	5795_EF335	*S. hominis*	Lancashire	Feb-16	pen ox ery clin	6	*mecD*
22	5787_EF169	*Staphylococcus warneri*	Lancashire	Feb-16	pen ox clin (trim)	48	*mecD*
23	5190_42462	*K. kristinae*	Sussex	Sep-15	pen ox clin	32	*mecD*
24	5794_EF323	*S. hominis*	Camarthenshire	Feb-16	ox clin tet (trim)	192	*mecD*
25	5798_EF375	*Staphylococcus haemolyticus*	Camarthenshire	Feb-16	pen ox ICR ery clin‡	8	*mecD*
26	5450_CC63A	*K. kristinae*	Ceredigion	Feb-16	pen ox clin tet	24	*mecD*
27	5800_EF393a	Unidentified	Pembrokeshire	Feb-16	pen ox (clin)	24	*mecD*
28	5799_EF381	Unidentified	Pembrokeshire	Feb-16	pen ox tet (clin)	32	*mecD*
29	5785_EF123	*S. hominis*	Wiltshire	Sep-15	pen ox ery clin	48	*mecD*
30	5198_3_76	Unidentified	Shropshire	Sep-15	pen ox	16	*mecD*
31	5788_EF188	Low discrimination (*S*. *warneri*/*S*. *hominis*	Shropshire	Feb-16	pen ox	16	*mecD*
32	5456_3–46	Unidentified	Shropshire	Sep-15	pen ox ery clin (trim)	192	*mecB*

*Refers to geographical locations shown in Fig. 2.

†Generated by Vitek2 AST-P634 card testing against cefoxitin screen (fox), benzylpenicillin (pen), oxacillin (ox), gentamicin, ciprofloxacin, erythromycin (ery), clindamycin (clin), linezolid, daptomycin, teicoplanin, vancomycin, tetracycline (tet), fusidic acid (FA), chloramphenicol, rifampicin and trimethoprim (trim). Also tested for inducible clindamycin resistance (ICR). Only resistances are shown, parentheses indicate intermediate resistance.

‡Indicates clindamycin resistance due to ICR.

§Indicates that the *mec* gene is carried on a plasmid.

Cefoxitin disc diffusion and Etest using the criteria for coagulase-negative staphylococci confirmed all 33 isolates to be methicillin-resistant. Cefoxitin MICs ranged from 6 to >256 mg l^−1^, Table 1. In agreement with these results, all 33 isolates were also resistant to oxacillin when tested by Vitek2. By contrast, all but one isolate was negative in the Vitek2 cefoxitin screen. The majority of isolates (25 out of 33) showed resistance to non-β-lactam antimicrobials using Vitek2 as follows: clindamycin (15 isolates), tetracycline (15), erythromycin (10), fusidic acid (5), with two isolates showing inducible clindamycin resistance, Table 1.

### Methicillin resistance determinants

*mecA* and *mecC* were not detected in the genome sequences of any of the 33 methicillin-resistant *M. caseolyticus*. Rather, isolates were positive for either *mecB* (six isolates) or *mecD* (27 isolates), Table 1. All *mecD*-positive isolates carried *mecD* within a *mecD-mecR1_m_–mecI_m_* gene complex, which appeared to be sited on the chromosome and shared 100 % nucleotide identity with the *mecD* gene of *M. caseolyticus* IMD0819. *mecB* was present in a *mecB* gene complex containing, *blaZ*_m_–*mecB–mecR1_m_–mecI_m_* associated with Tn*6045* and appeared to be plasmid-encoded in all isolates except in the case of 5456_3-46 where it is located on the chromosome. PCR and amplicon sequencing was used to confirm the chromosomal location of the *mecB* gene of 5456_3-46, showing it to be located downstream of the *orfX* gene. This is consistent with the chromosomal location of the *mecB* gene of *M. caseolyticus* JCSC7096 [21] (data not shown). All the *mecB* genes putatively associated with plasmids shared 99.9 % identity with the *mecB* gene of *M. caseolyticus* JCSC5402, this divergence being caused by a single nucleotide transversion, T754G. The *mecB* gene of 5456_3–46 shared 100 % nucleotide identity to the *M. caseolyticus* JCSC5402 counterpart. The best Blast hits for the five putative plasmid contigs carrying *mecB* were *S. aureus* 70774229 pSAWWU4229_1 followed by *M. caseolyticus* JCSC5402 plasmid pMLCC2.

### Phylogenetic and geographical relationships

**Fig. 1.**
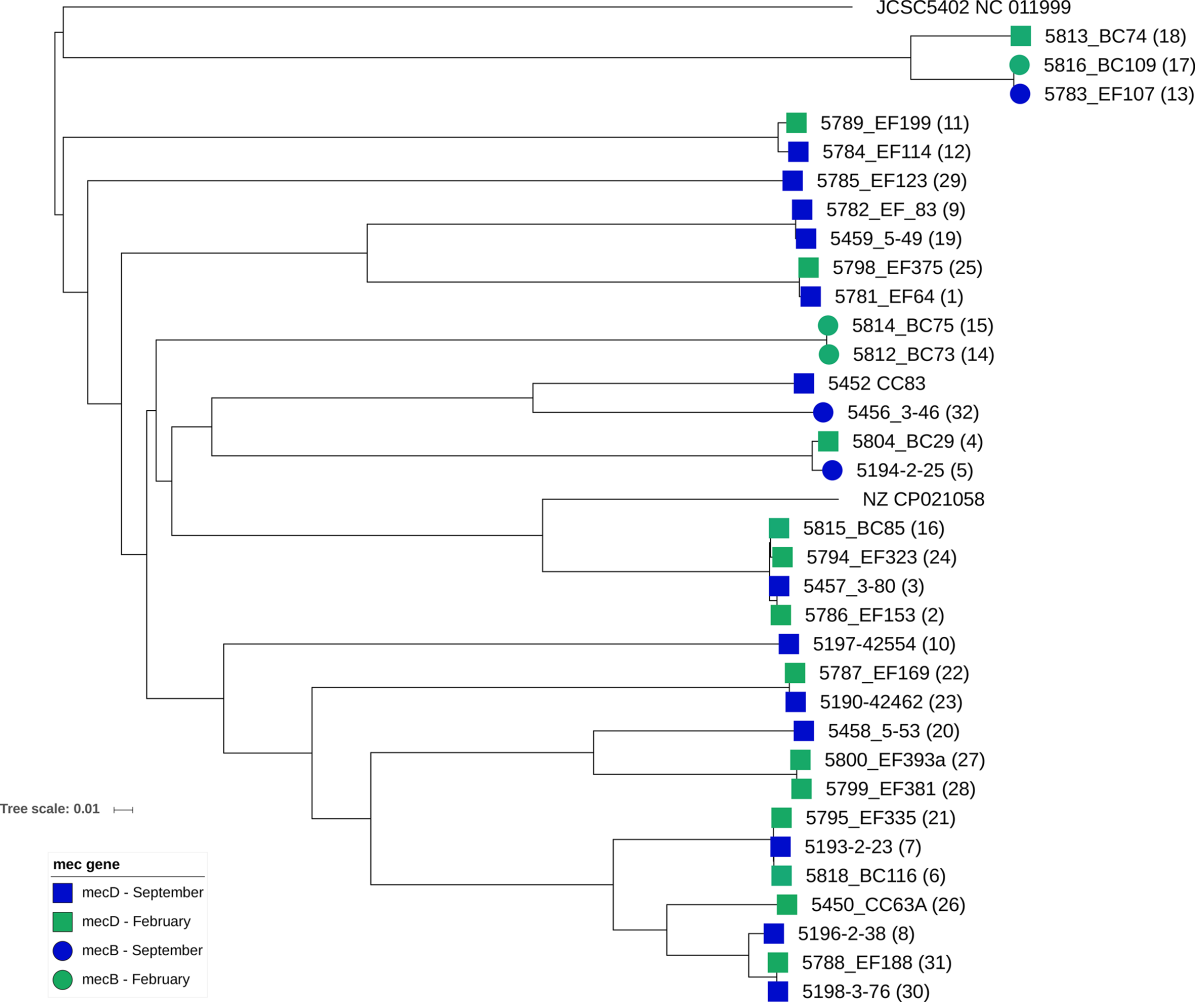
Phylogenetic relationships among methicillin-resistant *M. caseolyticus*. Neighbour-joining phylogeny generated using a core and accessory genome multilocus sequence typing scheme produced from all 35 isolates examined in this study and consisting of 1550 gene targets. In addition to the 33 isolates collected in this study, Table 1, the previously sequenced *M. caseolyticus* IMD0819 (NZ_CP021058.1) and JCSC5402 (NC_011999) are included. Numbers within brackets refers to geographical locations shown in Fig. 2.

The phylogenetic relationships between isolates was assessed using a cgMLST and accessory genome scheme consisting of 1550 gene targets generated from all 35 genomes in this study, Fig. 1. This showed that the 33 study milk isolates of methicillin-resistant *M. caseolyticus* represented a diverse population with a mean pairwise distance of 1075.4 allelic differences, Fig. 1. Both *mecB* and *mecD* were present in diverse isolates.

**Fig. 2.**
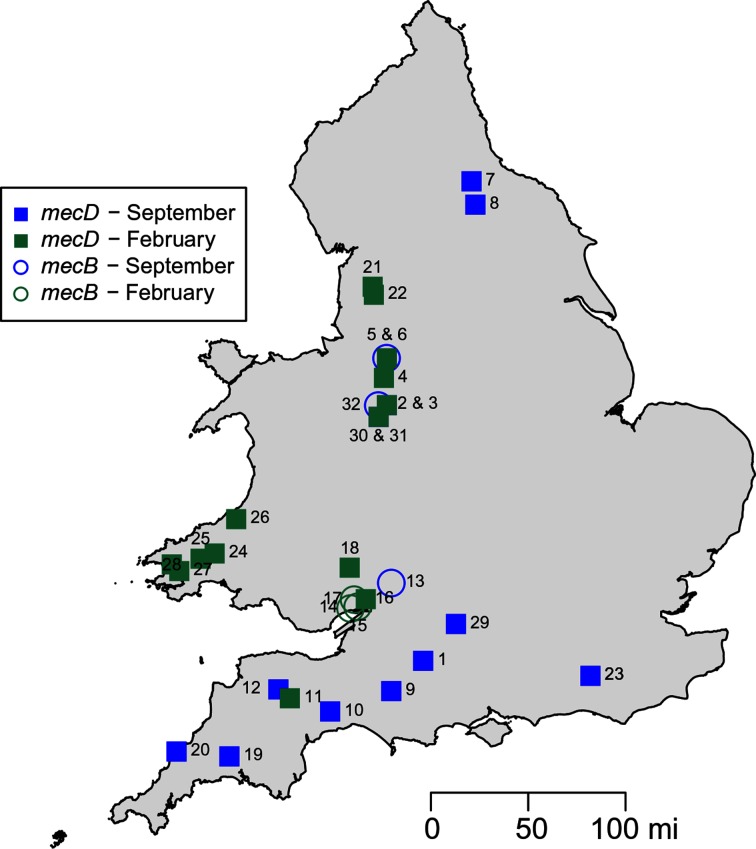
Geographical distribution and *mec* gene content of methicillin-resistant *M. caseolyticus*.

Location data showed that the isolates originate from 16 counties spread across England and Wales, Fig. 2. There were three instances of two different isolates being isolated from the same farm. Isolates 5198_3_76 and 5777_EF188 were isolated from the same farm in September 2015 and February 2016 respectively. As were 5786_EF153 and 5457_3_80. In both cases, the paired isolates carried the same *mec* type as each other, shared an identical antibiogram and were closely-related at the genome level. The first pair of isolates differing by only two alleles and the second pair by five alleles among the 1550 examined. In contrast, despite being isolated from the same farm, isolates 5194-2-25 and 5818_BC116 carried *mecB* and *mecD* respectively and were distantly related, differing by 1066 alleles. There were several cases of isolates from different farms in geographical proximity being highly-related, strongly suggestive of local dissemination between farms. For example, isolates 5812_BC73 and 5814_BC75 differed by only three alleles and originated from farms approximately 3.5 miles apart. This was not always the case with instances of proximal isolates being distantly related and closely related isolates coming from distal locations. For example, 5795_EF335 and 5787_EF169 differed by 704 alleles but were isolated from dairy farms approximately 4 miles apart. In contrast, despite being isolated approximately 213 miles apart 5190_42462 and 5787_EF169 were separated by only ten alleles. The most closely related *mecB* and *mecD* isolates where 5804_BC29 and 5194_2_25 which were isolated approximately 10 miles apart and differed by only 30 alleles.

## Discussion

Macrococci are becoming increasingly recognised as veterinary pathogens in their own right and as a potential reservoir of methicillin-resistance determinants that may transfer to the closely-related but more pathogenic staphylococci. Here we have isolated and characterised isolates of methicillin-resistant *M. caseolyticus* from dairy cattle bulk tank milk from England and Wales. All 33 isolates were confirmed as methicillin-resistant by cefoxitin disc diffusion, Etest and by Vitek2 oxacillin testing. However, only a single isolate was resistant in the Vitek2 cefoxitin screen. This raises the potential for these isolates to produce confusing susceptibility results and potentially for PBP2a-mediated resistance to be overlooked. The basis for this discrepancy in susceptibility is not clear and not often seen in *mecA* MRSA isolates, which are typically resistant to both oxacillin and cefoxitin according to Vitek [22]. This may relate to differences in the biochemistry of the proteins encoded by *mecB* and *mecD* compared with those encoded by *mecA*. Indeed, *mecA-* and *mecC-*encoded-proteins and strains behave differently [22, 23]. Alternatively, it may relate to the species background. For instance, cefoxitin is considered more reliable than oxacillin for the disc diffusion susceptibility testing of *mecA* MRSA [24], while in the case of *mecA Staphylococcus pseudintermedius* and *Staphylococcus schleiferi* oxacillin is recommended [25, 26]. The relatedness of macrococci to staphylococci may also present a diagnostic misreporting problem, with the former potentially being reported as the latter where molecular tests such as 16S rDNA sequencing are not used. Indeed, ten of the isolates in this study were identified as staphylococci by Vitek2 albeit we recognise that this system does not claim to identify *M. caseolyticus*. However, this is still problematic given the widespread use of Vitek2 and that such a result would be unlikely to be questioned. It is very possible therefore than *M. caseolyticus*, including methicillin-resistant isolates, are being overlooked in microbiology laboratories.

All isolates were positive for either *mecB* or *mecD* with the latter being the more frequent. This is the first report, to the best of our knowledge, of these methicillin-resistance determinants being found in Great Britain. Isolates came from a diverse bacterial population and were spread widely over England and Wales. Unfortunately, we cannot comment with confidence on*﻿*﻿ the prevalence of methicillin-resistant *M. caseolyticus* in the study area. Approximately 1100 bulk tank milk samples were assayed but our sampling approach, with convenience sampling and lack of full location data, mean this is not a statistically random sample nor can the exact number of duplicate samples be determined. Nonetheless, the isolate collection and associated data present a valuable and novel insight into the genomic epidemiology of methicillin-resistant *M. caseolyticus.* Future work should look at the prevalence of methicillin-resistant *M. caseolyticus*, not only in milk but in meat, where it can also be found, and microbiology laboratories, especially those dealing with mastitis samples should be mindful of its potential occurrence and possible difficulties in separating it from related staphylococci.

The presence in this study of methicillin-resistant *M. caseolyticus* with *mecB*-encoding plasmids is particularly worrisome given the description of a human MRSA isolate carrying a *mecB* plasmid which was probably acquired from macrococci. This highlights the potential role of animal microflora as a genetic reservoir for antimicrobial resistance genes and the need for a holistic approach to understanding the epidemiology of resistance determinates which does not just focus on pathogens. Furthermore, *mecB*/*mecD* MRSA isolates may cause a very major error in susceptibility testing, with resistant isolates being identified as susceptible on the basis of *mecA*/*C* PCR or PBP2a detection. Plasmid-mediated methicillin resistance may also have the potential to lead to the rapid dissemination of resistance among *S. aureus* and other staphylococci. For these reasons there is a need for laboratories to be aware of such isolates and for their potential spread to be carefully monitored. As for the potential transfer of *mecB* plasmids or other genetic elements between macrococci and staphylococci, there is little data, at present, on the mechanisms and frequency of such horizontal gene transfer between the two genera.

In our study, where the same farm was sampled twice, 4 months apart, we find persistence of related clones over that period. We also show that distantly related isolates with different *mec* genes can be found on the same farm at different time points. We show evidence for both the local and more distant spread of related isolates and also that highly-related isolates may carry either *mecB* or *mecD*, suggesting independent acquisition of both by a particular *M. caseolyticus* clone.

While these isolates came from bulk tank milk and thus no link to bovine mastitis can be apportioned here, methicillin-resistant *M. caseolyticus* has been isolated from bovine mastitis previously [3]. Together with our data, this supports a need for increased awareness of the potential role of *M. caseolyticus* in this economically important infection and the potential emergence of *mecB-* and *mecD*-mediated resistance in disease isolates.

To conclude, we report that a diverse population of methicillin-resistant *M. caseolyticus* encoding *mecB*/*mecD* are widely distributed in the dairy herd in England and Wales. This has implications in veterinary microbiology, particularly in light of potential difficulties with their identification and susceptibility testing. The risk and consequences of *mecB*/*D* spreading to *S. aureus* further underscore the need to better understand the epidemiology and antimicrobial resistance of *M. caseolyticus*. At present this is very poorly characterised and the data and genomes reported here are a valuable step in addressing this knowledge gap.

## Data bibliography

1. All genome sequences generated in this study have been deposited in Genbank under Bioproject PRJNA420921, study accession number SRP126085. Accession numbers for individual isolates (Biosample, SRA and Assembly) are provided in Table S1.

## Supplementary Data

Supplementary File 1
