# Supplementary material for: Genome analysis of methicillin resistance in Macrococcus caseolyticus from dairy cattle in England and Wales
Source: Microb Genom. 2018 Jun 19;4(8):e000191. doi: 10.1099/mgen.0.000191 (PMC6159548; doi:10.1099/mgen.0.000191)
Supplement: Supplementary File 1 [file mgen-5-191-s001.pdf]

Supplementary Table 1 Nucleotide accession details for isolates genome sequenced in this study. All sequences have been deposited under Bioproject PRJNA420921, study accession SRP126085.

Isolate	Biosample Accession	SRA Accession	Assembly Accession
5190_42462	SAMN08117945	SRR6344650	PIWJ00000000
5193_2_23	SAMN08117946	SRR6344651	PIWK00000000
5194_2_25	SAMN08117947	SRR6344648	PIWL00000000
5196_2_38	SAMN08117948	SRR6344649	PIWM00000000
5197_42554	SAMN08117949	SRR6344654	PIWN00000000
5198_3_76	SAMN08117950	SRR6344655	PIWO00000000
5450_CC63A	SAMN08117951	SRR6344652	PIWP00000000
5452_CC83	SAMN08117952	SRR6344653	PIWQ00000000
5456_3_46	SAMN08117953	SRR6344656	PIWR00000000
5457_3_80	SAMN08117954	SRR6344657	PIWS00000000
5458_5_53	SAMN08117955	SRR6344671	PIWT00000000
5459_5_49	SAMN08117956	SRR6344672	PIWU00000000
5781_EF64	SAMN08117957	SRR6344669	PIWV00000000
5782_EF 83	SAMN08117958	SRR6344670	PIWW00000000
5783_EF107	SAMN08117959	SRR6344675	PIWX00000000
5784_EF114	SAMN08117960	SRR6344676	PIWY00000000
5785_EF123	SAMN08117961	SRR6344673	PIWZ00000000
5786_EF153	SAMN08117962	SRR6344674	PIXA00000000
5787_EF169	SAMN08117963	SRR6344678	PIXB00000000
5788_EF188	SAMN08117964	SRR6344679	PIXC00000000
5789_EF199	SAMN08117965	SRR6344663	PIXD00000000
5794_EF323	SAMN08117966	SRR6344662	PIXE00000000
5795_EF335	SAMN08117967	SRR6344661	PIXF00000000
5798_EF375	SAMN08117968	SRR6344660	PIXG00000000
5799_EF381	SAMN08117969	SRR6344667	PIXH00000000
5800_EF393a	SAMN08117970	SRR6344666	PIXI00000000
5804_BC29	SAMN08117971	SRR6344665	PIXJ00000000
5812_BC73	SAMN08117972	SRR6344664	PIXK00000000
5813_BC74	SAMN08117973	SRR6344659	PIXL00000000
5814_BC75	SAMN08117974	SRR6344658	PIXM00000000
5815_BC85	SAMN08117975	SRR6344668	PIXN00000000
5816_BC109	SAMN08117976	SRR6344680	PIXO00000000
5818_BC116	SAMN08117977	SRR6344677	PIXP00000000
